# Ultrafast Micrometric Laser Structuring of Polymer Surfaces for Highly Organized Skeletal Muscle Tissue Modeling

**DOI:** 10.1002/advs.78129

**Published:** 2026-10-04

**Authors:** Lucas Duvert, Lorena Garcia‐Castillo, Clarissa Murru, Adrien Casanova, Daniel Ferry, Pierre Perrin, Solene Coste, Anne Bigot, Anne‐Patricia Alloncle, Frederique Magdinier, Stefano Testa

**Affiliations:** ^1^ Marseille Medical Genetics, MMG Aix‐Marseille University INSERM Marseille France; ^2^ National Institute of Optics National Research Council (INO‐CNR) Largo Enrico Fermi Firenze Italy; ^3^ LP3, Aix‐Marseille University CNRS Marseille France; ^4^ CINaM Aix‐Marseille University CNRS Marseille France; ^5^ Institut De Myologie Centre de recherche en myologie, Sorbonne Université INSERM Paris France

**Keywords:** biomimetic surface, direct laser writing, polymer structuring, skeletal muscle, tissue engineering, topographical guidance

## Abstract

Engineered surface topographies have proven to be an efficient method to replicate in vitro the architecture of highly organized tissues by controlling the spatial arrangement of cells. In this work, we studied the ability to guide muscle cell development using femtosecond‐laser‐induced topography on standard culture plates. This technique allows the creation of highly porous microstructured channels that exhibit strong hydrophobicity while offering mechanical cues for cell guidance. We characterized the resulting engineered surfaces by assessing their capacity to direct cellular organization. Myoblasts cultured on laser‐structured (LS) surfaces generated more mature myotubes exhibiting enhanced alignment and elongation compared with those cultured on standard plates. We applied the LS surfaces as a proof‐of‐concept approach to characterize myotube morphology in two common myopathies, Duchenne muscular dystrophy and facioscapulohumeral muscular dystrophy. The superior organization of the myotubes enabled a more accurate assessment at the single‐fiber level, revealing disease‐specific phenotypes. Here, we present the characterization and validation of an accessible, rapid, and reliable laser‐based method for generating micrometric topographies on standard culture plates tailored to skeletal muscle. The effectiveness and versatility of LS supports provide initial evidence for their use in disease modelling and support their future application in therapeutic screening and personalized medicine.

## Graphical abstract

Standard cell culture supports were functionalized by laser‐structuring to generate advanced topographies tailored to skeletal muscle tissue. The physicochemical modifications of the polymeric surfaces and their interactions with cells were thoroughly investigated, confirming both safety and effectiveness in guiding muscle cell organization. Finally, these functionalized supports were applied to characterize myotube morphology in two of the most common myopathies.

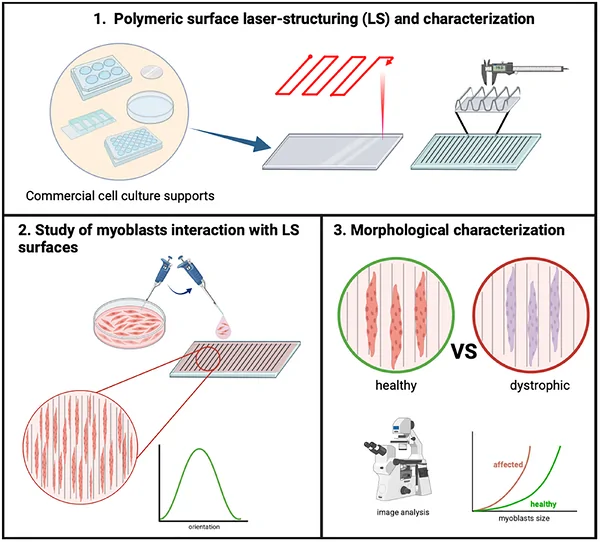

## 1 Introduction

Effective in vitro reproduction of tissues and organs requires a deep understanding of their respective cellular components and relative involvement in maintaining tissue homeostasis [1]. Added to the reproduction of tissue function, these prerequisites constitute a critical stage in tissue engineering [2]. Yet, biological complexity is not the only issue to be addressed for in vitro modeling [3]. The specific spatial organization of the different cell populations and the replication of the typical tissue/organ architecture are as important as the biological counterpart to obtain a reliable in vitro model [4]. In this regard, skeletal muscle tissue is characterized by an anisotropic structure of aligned muscle fibers fundamental for the isometric or eccentric contractility and transmission of effective force [5].

Nowadays, several approaches aim to mimic tissue‐specific architecture in both 2D and 3D, mainly by replicating the organization of the extracellular matrix (ECM) [6]. In this context, various tissue engineering strategies can either replace or be implemented in traditional 2D cell culture to achieve higher cell organization [7, 8]. Among these methods, the most widely used for its accessibility and easy workflow integration is the addition of 2D/3D topographical cues to the surface of cell culture supports [9]. Micropatterned surfaces have indeed proven to be a suitable approach for modeling different tissues, from endothelium [10] to neural [11] and muscle tissue [12, 13]. For the latest, micropatterned surfaces are able to influence myoblast orientation, differentiation, and maturation [14, 15]. Photopatterning combined with soft lithography and melt‐casting systems constitute the two most widespread surface micropatterning methods used to create micro‐grooved and topographical modifications on natural or synthetic polymer surfaces [14, 16]. Both these methods rely on the use of a master mold, which defines the geometry and characteristics of the patterning. Due to the multi‐step manufacturing process involved in these techniques, the commercially available systems remain expensive to integrate into traditional cell biology workflows and can yield variable results depending on the brand and cell types to be used [17, 18].

Laser processing is a well‐known non‐contact approach for micro‐to‐nano structuring materials for various applications. Laser irradiation with pulses ranging from nanoseconds down to femtoseconds can be used for surface texturing through direct laser ablation or via the creation of self‐organized structures [19]. Complex and tailored patterns with multiscale characteristics can be produced with high precision by scanning a focused beam on the targeted material surface [20]. This method therefore stands out from mold‐based systems by its efficiency and versatility, allowing the pattern geometry to be reconfigured in real time through software‐controlled parameter adjustments, rather than requiring the fabrication of dedicated molds for each new design. Ultrashort laser pulses (in the picosecond or femtosecond range) induce very high laser intensity in the material, leading to strong nonlinear, intensity‐dependent absorption (ionization mechanisms). The absorbed energy is then transferred to the lattice, leading to thermomechanical relaxation and matter ejection [21]. In this manner, a wide range of materials can be structured by direct laser ablation regardless of their optical or electrical properties. These mechanisms have been widely investigated theoretically and experimentally for dielectric materials [22, 23]. Beyond a certain threshold, absorption mechanisms lead to sufficient energy deposition to drive the ablation of the material. Additionally, the ultra‐short pulses are shorter than the heat diffusion time in the surrounding lattice (>10 ps), leading to a minimal heat‐affected zone [24].

In this context, the creation of bioinspired laser‐structured (LS) surfaces has been increasingly studied [19, 25, 26] for their efficiency and ability to process a wide range of synthetic and natural materials. Similarly to photopatterning and melt‐casting methods, the LS surfaces are able to influence cell behavior and intrinsic mechanisms such as adhesion, proliferation, and alignment [27, 28, 29], creating, for example, an environment conducive to cell interaction and osteogenic differentiation [30].

For these reasons, we choose here to exploit the properties of femtosecond lasers for structuring the polymeric surfaces of commercially available cell‐adhesion‐treated culture plates (standard plates). The laser irradiation of polystyrene (PS), the most widespread polymer used for cell biology labware, was thus thoroughly investigated. The physical characterization and cytotoxicity of the resulting LS substrates were first evaluated. Second, the ability of the LS surface to drive myoblast organization and promote their differentiation was studied.

Finally, we investigated the potentialities of LS surfaces in generating advanced 2D muscle models, validating them through comprehensive screening based on image analysis. In addition, two of the most common muscular dystrophies, Duchenne muscular dystrophy (DMD) and Facioscapulohumeral dystrophy (FSHD), were modelled using human immortalized myoblasts. DMD is an X‐linked muscle‐wasting disease that affects 1: 5000 male individuals with lethal consequences due to respiratory and cardiac complications [31]. FSHD is an autosomal dominant disease affecting specific facial, shoulder, and pelvic girdle muscles, causing progressive weakness. The incidence rate is 1: 20,000, making FSHD the third most common myopathy. Especially for FSHD, existing animal and cellular models cannot fully recapitulate the pathophysiological processes, highlighting the need for reliable disease models based on multidisciplinary approaches [32].

In conclusion, we present here a fast laser‐based, single‐step, and chemical‐free alternative to generate micrometric topographies tailored for skeletal muscle modeling on standard polystyrene cell culture plates. The efficiency of LS plates in driving muscle cell organization and differentiation can improve our understanding of skeletal muscle behavior in both physiological and pathological contexts, while providing an accessible tool for high‐throughput screening. The versatility of this approach enables the generation of LS patterns of virtually any geometry and can therefore be applied to model a wide range of tissues and organs.

Widespread dissemination of this methodology would allow standard culture plates to be readily functionalized into tailored micro‐structured supports, offering an accessible and effective strategy to advance conventional 2D cell‐culture systems.

## 2 Materials and Methods

### 2.1 Micrometric Laser Structuring

#### 2.1.1 Laser Topography and Optical Characterization

Generation of the topography was carried out on cell‐adhesion‐treated culture plates (standard plates, Thermo Fisher) using a pulsed picosecond‐to‐femtosecond laser (Light Conversion Carbide CB‐01 Yb: KGW) with a fundamental wavelength of 1030 nm at a frequency of 60 kHz. The laser's average power is 5 W, and its maximum energy per pulse is 85 µJ at 60 kHz. A polarizer coupled with a half‐wave plate is positioned in the optical path of the laser to tune the energy deposited on the substrate. The laser beam is finally routed on a computer‐controlled high‐speed optical scan system (ScanLab intelliSCAN14) coupled with a 70 mm focal length F‐Theta lens (Linos F‐Theta‐Ronar lenses), which allows us to generate complex marking patterns in a fast and efficient manner, covering a maximum field of 50×50mm^2^. The waist of the beam has been estimated using a regression method [33]: 2wo = 36 µm.

To characterize and control the dimensions of the created channels, the influence of pulse duration, energy, and overlap was investigated together with the influence of channel depth and spacing on cell alignment. The physical characterization of the laser‐structured surfaces was achieved using scanning electron microscopy (SEM, JEOL JSM‐6390, Tokyo, Japan) working at 3 kV after metalization (gold, 20 nm by sputtering method) of the sample. For all the parameters considered, channel depth and width were evaluated by confocal microscopy (Keyence, VK‐X3000) through an average of 30 profile lines taken perpendicularly to the channel orientation. The depth value is calculated between the highest and the lowest point encountered on the profile line.

#### 2.1.2 Contact Angles

Wettability modifications of the LS plates were characterized by contact angle measurement. Five microliters of distilled water were deposited on standard and LS surfaces, and photographed at two different angles (0° and 90°) through a camera (DAHUA) equipped with a 10X lens (Computar). The droplets were illuminated by a white light source (Wild HeerBrugg) placed on the opposite side of the camera to obtain sharp images of their edges. The angle between the droplet and the surface was calculated using the *Contact Angles* plugin for FIJI software. The obtained value was used as an index of hydrophobicity: values ranging from 90° to 0° indicate an increase in hydrophilicity, while values from 90° to 180° indicate an increase in hydrophobicity. Top pictures of the droplets were taken using numerical microscopy (Keyence VHX‐7000).

#### 2.1.3 Chemical Analysis

The surface chemistry and thermal behavior of standard and LS plates were recorded in attenuated total reflectance (ATR, germanium crystal) mode using a Bruker VERTEX70 mid‐IR Fourier Transform spectrometer equipped with a temperature‐stabilized DLaTGS detector. All spectra were accumulated on the surface of the different areas in a spectral range of 650–4000 cm^−1^ by recording 200 scans at a resolution of 4 cm^−1^. The spectra were normalized to the maximum absorbance reached.

#### 2.1.4 Cell Culture

Murine myoblasts C2C12 (ATCC) were cultured according to a previously published protocol [34] in T‐75 Flasks (Sarstedt) at 37°C and 5% CO_2_ in Dulbecco's modified Eagle medium GlutaMAX (DMEM, Gibco) supplemented with 10% heat‐inactivated fetal bovine serum (FBS, EuroClone), penicillin (100 IU mL^−1^, Gibco), and streptomycin (100 mg mL^−1^, Gibco). Once 90% confluency was reached, cells were transferred to a differentiation medium (DM) composed of DMEM supplemented with 2% horse serum (Gibco). DM was changed every 48 h for up to 1 week to induce final differentiation.

Human immortalized myoblasts were obtained through a collaborative agreement with the Myoline platform (Institute of Myology, Paris). Cells were cultured in Growth Medium (GM) composed of 4 parts of DMEM GlutaMAX (Gibco) and 1 part of Medium 199 (Gibco), supplemented with 20% of FBS (Euroclone), Fetuin (25 µg/mL, Life Technologies), hEGF (5 ng/mL, Life Technologies), bFGF (0.5 ng/mL, Life Technologies), Dexamethasone (0.2 mg/mL, Sigma) and insulin (5 µg/mL, Sigma). Once 90% confluency was reached, cells were transferred to DM composed of DMEM GlutaMAX (Gibco) supplemented with insulin (10 µg/mL, Sigma). DM was changed every 48 h for up to 1 week to induce final differentiation.

#### 2.1.5 Proliferation Assay

C2C12 cells were seeded onto standard and LS 12‐well plates (Thermo Fisher) at 5 × 10^4^ cells per well. After 24, 48, and 72 h of culture, cell viability and proliferation were determined by the MTT (3‐(4,5‐dimethylthiazol‐2‐yl)‐2,5‐ diphenyltetrazolium bromide) colorimetric assay (Invitrogen, Thermo Fisher Scientific). This test is based on the reduction of the tetrazolium salt by the mitochondria of living cells. The absorbance measured at 600 nm is proportional to the number of living cells.

#### 2.1.6 Immunostaining Analysis

Samples were analyzed by immunofluorescence according to the following protocol: cells were fixed with a solution of 4% Paraformaldehyde (Thermo Scientific) at 4°C for 10 min, then rinsed twice with Phosphate Buffered Saline (PBS, Gibco). Subsequently, cells were permeabilized in a 0.3% solution of Triton X‐100 (Sigma) in PBS for 1 h at room temperature (RT) and blocked with a blocking solution consisting of 10% natural goat serum (Thermo Fisher Scientific), 1% glycine (Thermo Fisher Scientific), 0.1% Triton X‐100 in PBS for 1 h at RT. Afterward, cells were incubated for 2 h at RT with primary antibodies diluted 1:100 in blocking solution: mouse monoclonal anti‐Myosin Heavy Chain (MF20, DHSB); mouse monoclonal anti‐Lamin A/C (Santa Cruz); rabbit polyclonal anti‐Titin (Proteintech); rabbit polyclonal anti‐Dystrophin (Invitrogen). After rinsing twice (15 min each) with a washing solution consisting of 1% Bovine Serum Albumin (BSA, Sigma) and 0.2% Triton X‐100 in PBS, cells were incubated for 1 h at RT with secondary antibodies diluted 1:200 in blocking solution: Alexa Fluor 555‐conjugated goat anti‐mouse IgG (H + L; Thermo Fisher Scientific) and Alexa Fluor 488‐conjugated goat anti‐rabbit IgG (H + L; Thermo Fisher Scientific). Finally, nuclei were counterstained with 300 nM DAPI (Thermo Fisher Scientific) in PBS for 10 min.

Images were taken with the Zeiss Axio Observer Z1/7 inverted fluorescence microscope and the Zeiss LSM 800 AiryScan confocal microscope, using ZEN microscopy software.

#### 2.1.7 Image Analysis

Different image analyses were performed to characterize the myotubes using the FIJI software. Myotubes boundaries were manually defined, as well as nuclei. The Fusion Index (FI), defined as the ratio of nuclei in Myosin Heavy Chain‐expressing myotubes (containing at least three nuclei) to the number of nuclei, was manually scored. Similarly, the distribution of nuclei per myotube, also referred to as the maturation index, was scored. Finally, other parameters such as the myotube area coverage, the myotube length, diameter, and the number of branches per myotube were manually calculated.

The orientation of the myotubes was studied using the *Orientation J* plugin. In particular, the *Orientation J distribution tool* allows us to obtain a weighted histogram of the orientation of the analyzed elements, ranging from 0 to 180 degrees. In contrast, the weight of this orientation is indicated by the coherency: a value ranging from 0 (isotropic areas) to 1 (highly oriented surfaces) calculated by the *Orientation J dominant direction tool* [35].

For 3D reconstructions, Z‐stack CZI images were elaborated with IMARIS software (Oxford Instruments, UK).

#### 2.1.8 RT‐qPCR

C2C12 cells were seeded in 6‐well plates (Thermo Scientific) at 2,5 × 10^5^ cells per well, with three wells for each condition (standard and laser‐structured) and each experimental time point (**d0** – day of differentiation induction, **d4, d7, d11** – day 4,7 and 11 of differentiation). RNA was extracted using the standard phenol‐chloroform method. 1 µg of RNA for each sample was reverse transcribed using the SuperScript IV reverse transcriptase (Invitrogen), according to the manufacturer's instructions. Glyceraldehyde 3‐phosphate dehydrogenase (GAPDH) was selected as an endogenous control after verifying its stable expression. Quantitative RT‐PCR analysis was performed with a QuantStudio 5 Real‐Time PCR System (96‐well, Applied Biosystems). Each cDNA sample was amplified using a LightCycler 480 SYBR Green I Master (Roche). Relative mRNA levels were calculated by the delta‐delta CT method (ΔΔCt) [36]. Primer specificity was confirmed by melting curve analysis.

Primers used are listed below:

1. GAPDH: Fw: ACTGAGCAAGAGAGGCCCTA; Rv: TATGGGGGTCTGGGATGGAA
2. MyoD1: Fw: GGAAGGGAAGAGCAGAAG; Rv: AAGGACTACAACAACAACAAC
3. Desmin: Fw: CGTGACAACCTGATAGAC; Rv: TGTTCTCTGCTTCTTCTC
4. MHC: Fw: AACTGAAGAATGCCTATGAAG; Rv: TCTGCTATCTCTTGTTCTAAGT
5. Myomaker: Fw: GTTGCTTCACTCTGTTCA; Rv: TATTTACTGGTCTAGGGTTCT
6. C‐met: Fw: AGTCCTATATTGATGTCTTAC; Rv: AACCTGATTATTCTTGTATGA

#### 2.1.9 Western Blotting

C2C12 cells cultured on standard or laser‐structured plates were collected at the same experimental time points used for RT‐qPCR analysis (d0, d4, d7, and d11). Cells were lysed using RIPA buffer (150 mm NaCl, 50 mm Tris‐HCl pH 8.0, 1% Triton X‐100, 0.5% sodium deoxycholate, 0.1% SDS) containing a protease and phosphatase inhibitor cocktail (100x; Thermo Scientific). Lysates were homogenized and centrifuged at 20,000 *g* for 20 min at 4 °C, and the supernatants were collected. Total protein concentration was determined using the Pierce BCA Protein Assay Kit (Thermo Scientific). Equal amounts of protein (10 µg) were heat‐denatured in LDS sample buffer (4×) containing NuPAGE antioxidant (Invitrogen), separated by SDS‐PAGE (4561086, Bio‐Rad), and transferred onto nitrocellulose membranes (1704159, Bio‐Rad). Membranes were blocked with 5% non‐fat dry milk in Tris‐buffered saline containing 0.05% Tween (TBS‐Tween) and then incubated overnight at 4°C with primary antibodies against Myosin Heavy Chain (MHC; 1:1000, Bio‐Techne MAB4470, monoclonal, clone MF20), Desmin (1:3000, Abclonal A3736, monoclonal, clone ARC0235), GAPDH (1:40000, Sigma MAB374, monoclonal, clone 6C5) and Tubulin (1:4000, Sigma T6199, monoclonal, clone DM1A). HRP‐conjugated anti‐mouse and anti‐rabbit IgG secondary antibodies (1:10000, Bio‐Rad) were subsequently applied. Membranes were washed three times for 5 min with TBS‐Tween following each antibody incubation. Protein bands were visualized using Clarity Western ECL substrate (170‐5061, Bio‐Rad) and detected with the ChemiDoc MP Imaging System (Bio‐Rad). Densitometric analysis was performed using Image Lab software (Bio‐Rad).

### 2.2 Statistical Analysis

All experiments were performed at least in biological triplicate (*n* = 3). Data were analyzed using GraphPad Prism 8, and values were expressed as means ± standard deviation. Statistical significance was tested using the appropriate statistical test. A probability of less than 5% (*p* < 0.05) was considered statistically significant.

## 3 Results

### 3.1 Topographical Modification of Polystyrene Culture Plates

Polystyrene (PS) culture plates (standard plates) are predominantly used as cell culture labware and were thus first used to evaluate the potentialities of laser‐induced topography. Given the absorption spectrum of PS, primarily situated in the UV around 260 nm, this material is transparent to the infrared wavelength of our laser. This issue was addressed using ultrashort pulses in the order of picoseconds to femtoseconds to maximize the interaction between the pulse and the bulk of the material via nonlinear absorption. Two distinct laser pulse durations accessible with our laser source, 10 ps and 213 fs, were thus considered to create the topography and further evaluated. The precise experimental set‐up for laser structuring is detailed in Figure S1. Parallel lines will be used to ensure the guiding and alignment of the cultured cells and myotube differentiation.

#### 3.1.1 Channel Characterization

For both pulse durations, pulse energy was investigated from 4.8 to 36.3 µJ, and the overlap of the pulses ranged from 48% to 95%, with 100% overlap corresponding to the complete superposition of two consecutive laser pulses. For this analysis, the channel spacing was set to 125 µm (Figure 1a).

**FIGURE 1:**
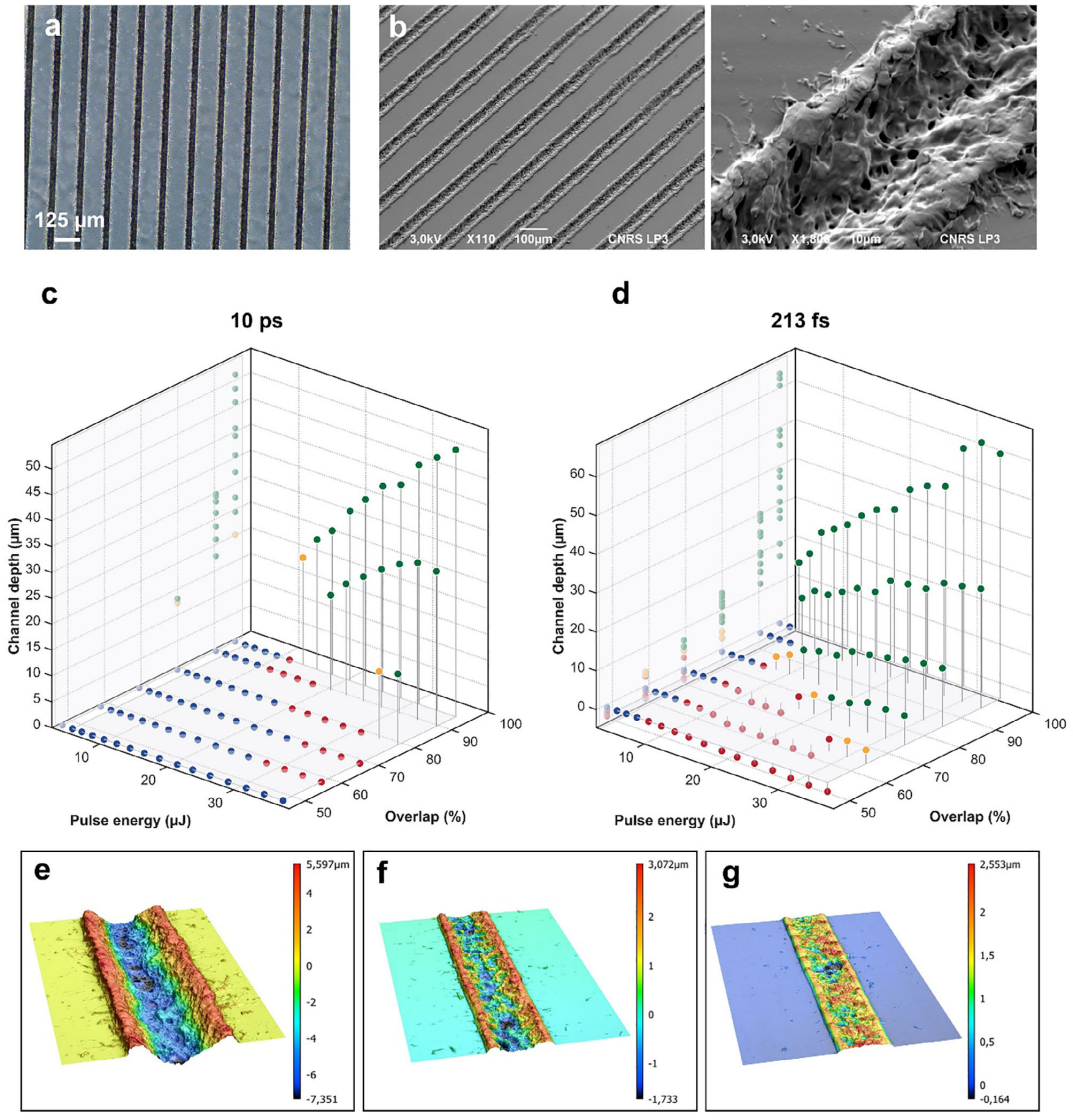
(a) Macroscopic observation of LS plates by optical microscope; channel spacing is set at 125 µm. (b) Scanning electron microscope (SEM) pictures. (c, d) Parametric study of the laser‐created channel depth depending on the laser pulse energy and the shot‐to‐shot overlap for 10 ps (Left) and 213 fs (Right). The depth values are projected on the z‐axis for easier reading. In addition to their depth values, laser‐induced channels can be distinguished by their general morphological appearance: green dots indicate well‐formed channels with pronounced topographical modifications and a high depth‐to‐protrusion ratio(**e**), yellow dots indicate the creation of porosity with a slightly indented surface and a lower depth‐to‐protrusion ratio (f), red dots indicate only the creation of porosities with a negative depth‐to‐protrusion ratio (g). Blue dots indicate the absence of marking by the laser pulses. The scale bar in panels (e– g) represents the depth (negative values) and height (positive values) of the channels relative to the plane of the polymer surface (value 0).

The observation of the resulting structured surfaces with a scanning electron microscope (SEM) revealed a highly organized pattern of channels with consistent results across the processed sample (Figure 1b). The interaction of the laser with the polymeric surface induced a partial and localized melting and vaporization of the material, altering the topography from a smooth surface to an irregular and porous one. The partial redeposition of the melted material could be noticed on the edge of the created channels. Confocal microscopy topographical analysis revealed that this material redeposition can account for almost 50% of the total height of the created topography. This amount logically varied depending on the energy density deposited on the substrate, either by a higher pulse energy and/or overlap. Based on the objectives of our work and their potential benefits in guiding cell organization, the depth measurement of the channels considered the creation of these protrusions (Figure S2). These depths are reported in Figure 1c,d for both pulse durations considered. They are divided into four categories (Figure 1c,d): reproducible channels of sufficient depths (Figure 1e), porous channels of limited depths (Figure 1f), both potentially suitable for cell driving and alignment, porous surfaces without any depth created (Figure 1g), and the absence of marking.

Femtosecond pulses offered a large number of energy/overlap couples suitable for the creation of channels with adjustable depth. For both parameters considered, the evolution of its complement was logically accompanied by a progressive decrease of the channel depths, going, for a large majority of them, through all four conditions previously described. The achievable channel depth varied from 6 µm to 65 µm, with an associated width varying from 30 (size of the laser beam) to 127 µm.

On the other hand, picosecond pulses presented fewer viable options: 20% of the conditions tested resulted in well‐defined channels, while femtosecond pulses reached 43%. Contrary to femtosecond ablation, we could also notice a clear marking threshold, beyond which not enough energy was deposited on the polymeric surface to induce topographical changes or create porosity. The range of achievable depth thus varied from 19 µm to 51 µm. Given poorer energy deposition due to limited nonlinear absorption, more energy was required with picosecond pulses to achieve the required intensity for breakdown [24]. This absorption difference was also visible in the channel porosity (Figure S3).

Based on these results, a pulse duration of 213 femtoseconds, an overlap of 79% (corresponding to a scan speed of 0.4 m/s), and a pulse energy of 22.8 µJ (fluence of 2.24 J/cm^2^) will be used for all the subsequent experiments. These parameters allowed the creation of 12.2 ± 0.96 µm deep channels of 43.9 ± 1.7 µm width. The protrusion of melted material accounted for 41% of the total height of the induced topography.

#### 3.1.2 Autofluorescence

Polymer surfaces are known to be autofluorescent, especially near the UV light spectrum. The LS surfaces displayed increased autofluorescence near UV light (DAPI emission peak, 454 nm), a phenomenon that decreased moving toward infrared light (CY5 emission peak, 670 nm, Figure S5e–h). To determine the role of the laser‐structuring process in the increase of PS autofluorescence, we analyzed the effect of stronger laser‐PS interaction through an incremental number of deposited laser pulses (1, 2, 3, 4, 6, 8, 10) on the PS surface (Figure 2). We noticed that one laser pulse only creates limited damage to the PS surface and is not enough to induce porosity (Figure 2a). By increasing the number of laser pulses, we observed an increase in porosity and damaged area following the laser interaction (Figure 2b–g,h). Linear regression analysis revealed a strong positive correlation between the number of laser pulses and the damaged area, with an R^2^ of 0.906 and a highly significant association (Figure 2k). Furthermore, a direct correlation was observed between the number of laser pulses and the fluorescence levels at the DAPI wavelength (R^2^ of 0.9262, Figure 2l). The latter was calculated as the Mean Gray Value (MGV) of a defined Region of Interest (ROI, yellow square, Figure 2i) surrounding the laser pulse interaction zone. Interestingly, when the fluorescence value was normalized to the damaged area (Figure 2j), the strength of the correlation with the number of laser pulses was substantially reduced (R^2^ = 0.4367, Figure 2m), indicating that the increase in fluorescence observed is closely associated with the increase in the damaged area.

**FIGURE 2:**
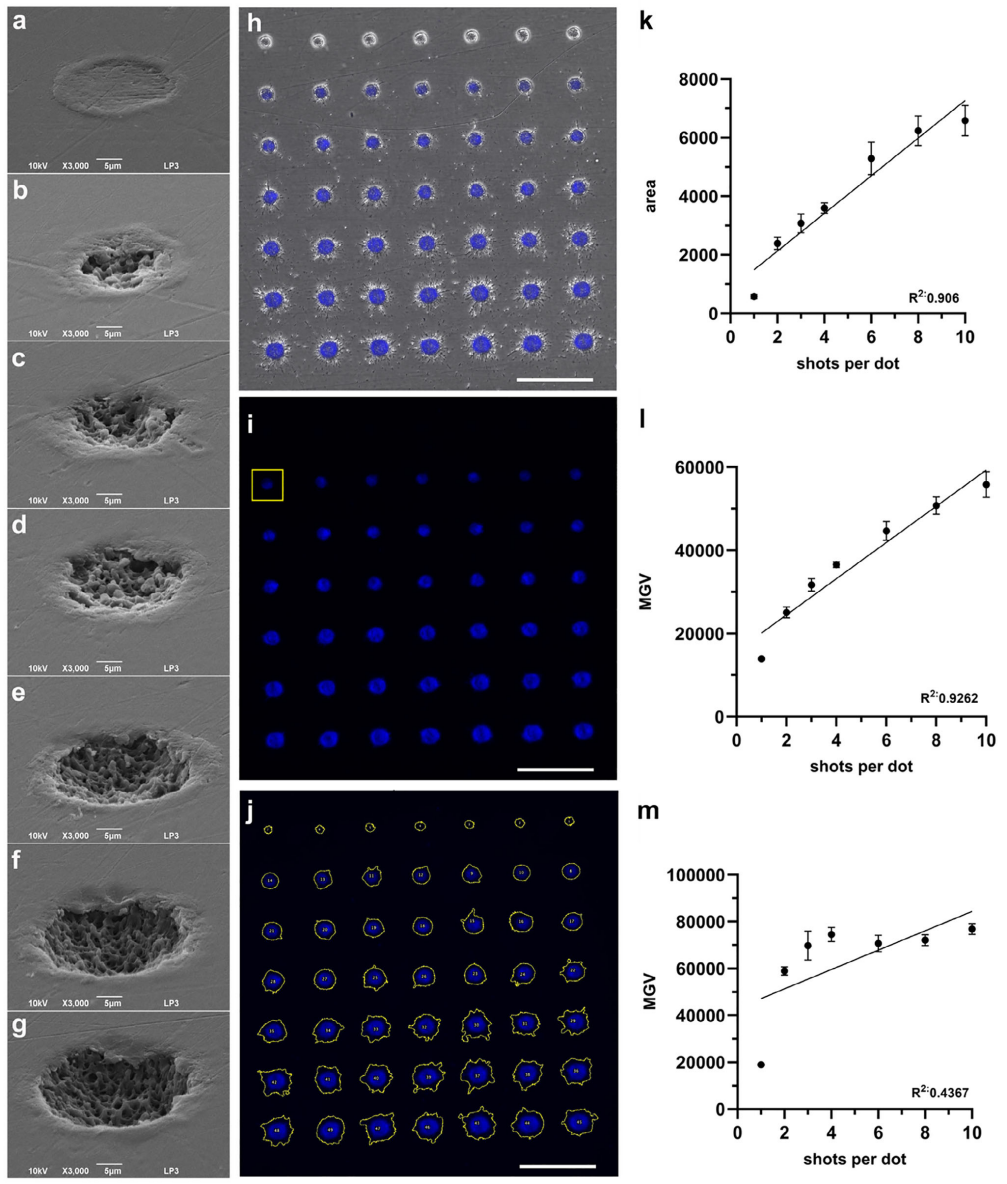
Systematic study of the effect of multiple laser shots on standard plates' autofluorescence. The effect of laser shots in modifying the porosity of the PS surface was studied by SEM for different numbers of pulses per spot: 1 (a), 2 (b), 3 (c), 4 (d), 6 (e), 8 (f), 10 (g). The area of the laser hit spots (h) was calculated and represented (k). The DAPI wavelength fluorescence levels at each condition (i) were calculated as MGV (Mean Grey Value) per ROI (Region of Interest) of 100×100 pixels (i, yellow square) and reported (l). The fluorescent particles were identified using the Fiji software (j), and the resulting MGV per particle is reported (m). For all experiments, *sample size n* = 7, *p* < 0.0001. Scale bar = 150 µm.

It has been widely demonstrated that exposing auto‐fluorescent material, biological or polymeric, to focused illumination can reduce the autofluorescence of the material itself [37, 38, 39]. In line with this, we observed that continuous exposure of LS surfaces under the DAPI wavelength of a fluorescence microscope (Zeiss Axio Observer Z1/7) led to a drastic reduction of the channel's autofluorescence under the observation spot (Figure S4a–l). To generalize this effect to the complete LS surface, a 3 W UV‐LED (Chanzon, spectrum centered at 365 nm) was chosen (Figure S5a,b). The choice of light source was motivated by the spectral overlap between the UV LED emission spectrum and the polystyrene absorption spectrum (Figure S5c,d). After measuring the reduction in MGV for the DAPI wavelength over time (Figure S5i), an irradiation time of 10 min was chosen, and the reduction in autofluorescence level was calculated for four commonly used wavelengths (DAPI, GFP, CY3, CY5). The analysis showed a drastic reduction in MGV levels for the two most autofluorescent fluorophore wavelengths (DAPI and GFP). CY3 and CY5, for which the autofluorescence level was already negligible (Figure S5j), were not affected by the UV LED exposure. A photobleaching step using UV‐LED was thus added immediately after the laser structuring process to reduce the LS surface autofluorescence.

#### 3.1.3 Contact Angles

Contact angle analysis was performed to characterize the wettability of the LS surfaces (Figure S6). The analysis was conducted on standard and LS plates, for four different LS channel spacings (75 µm, 100 µm, 125 µm, and 150 µm), using the previously established laser parameters.

This study revealed an increased surface hydrophobicity in the presence of laser‐induced channels, as demonstrated by the different shapes of the droplets deposited on standard and LS surfaces when photographed in parallel (0°, Figure S6b,c) or perpendicular (90°, Figure S6d,e) to the channels. For all tested spacings, the droplet stretched along the direction of the channel, which created a hydrophobic barrier, a hypothesis confirmed by the contact angle values measured in both directions (0° and 90°). Interestingly, while the angles measured under the standard condition (Figure S6b,d) exhibited two identical values (Figure S6h), all LS spacings yielded orientation‐dependent measurements, with differences reaching nearly 80% of the control angle (Figure 6h). This effect was made evident by the top‐view images of droplets on standard (Figure S6f) and LS (Figure S6g) surfaces, which revealed a marked impact on droplet sphericity caused by the laser‐induced channels.

### 3.2 Impact of Laser‐Structured Surface on Myoblast Behavior

#### 3.2.1 Biocompatibility: FTIR and MTT Assay

To verify whether the interaction of the laser with the PS surface could form cytotoxic compounds, standard and LS plates were analysed through ATR‐FTIR spectroscopy. As shown in Figure 3, the absorption peaks at the wavenumbers of 2942 and 2858 cm^−1^ can be attributed to the asymmetric and symmetric stretching vibrations of the CH_2_ methylene groups (saturated C─H). Furthermore, a notable absorption peak at 1460 cm^−1^ can be associated with the stretching vibrations of the aromatic C═C bonds, while the peak at the wavenumber of 725 cm^−1^ corresponds to the C─H out‐of‐plane bending vibration [40]. Interestingly, the curves of the two absorbance spectra overlapped, indicating the absence of side products due to laser‐PS interaction (Figure 3a).

**FIGURE 3:**
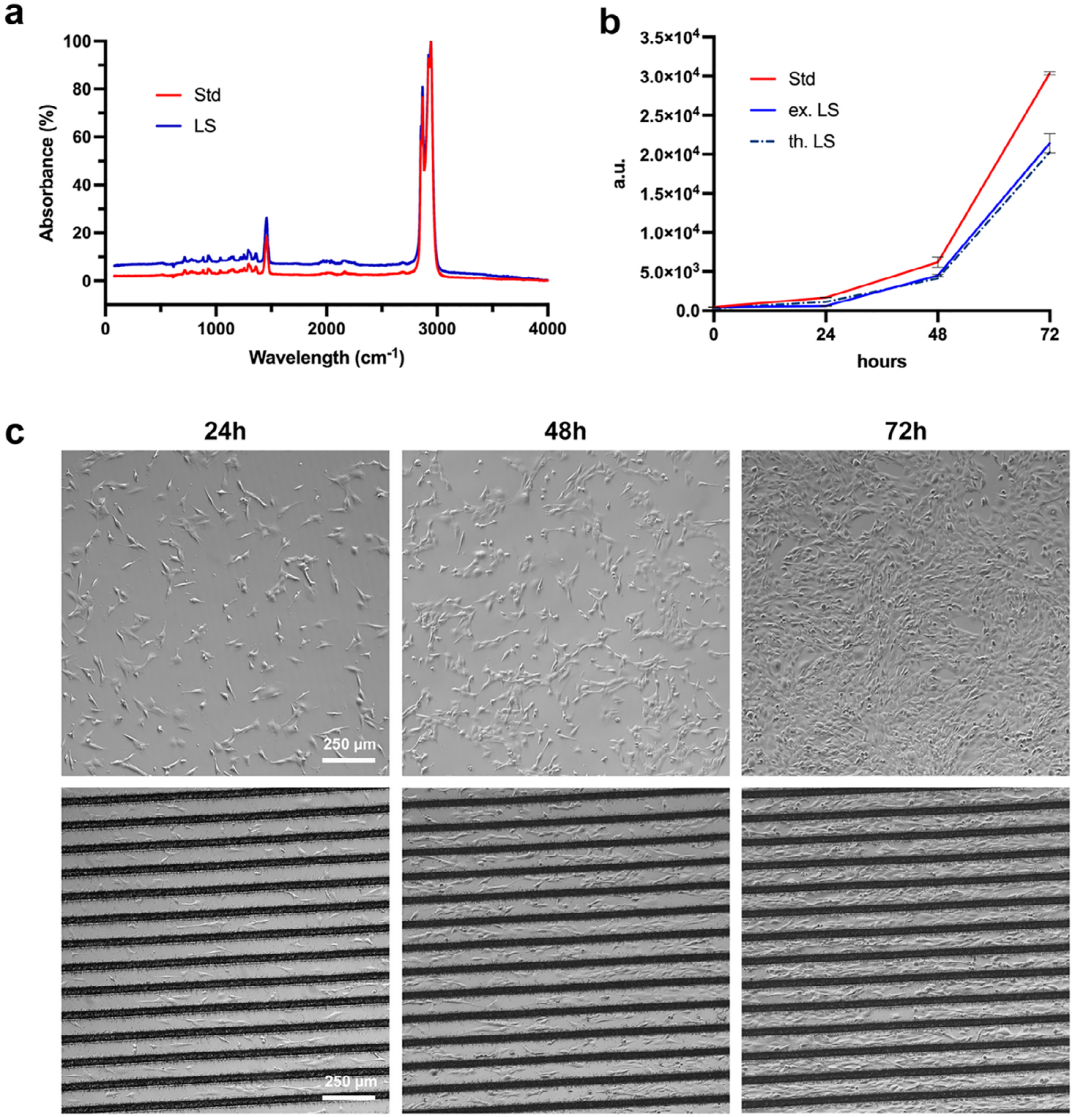
(a) Infrared spectra of the standard surface before (Std, red line) and after laser structuring (LS, blue line); (b) MTT assay. The C2C12 cell growth at three experimental time points (24 h, 48 h, and 72 h) is plotted for the cells seeded on a standard surface (Std, red line) and LS (ex. LS, blue line) surfaces. A theoretical curve was also plotted, taking into account the area available on the LS surface (th. LS, dashed dark blue line). (a.u. = arbitrary unit); (c) Brightfield pictures of the cells cultured on standard (top pictures) or LS (bottom pictures) surfaces at the three experimental time points (24 h, 48 h, and 72 h).

Secondly, to assess the effect of LS surfaces on cell viability and proliferation, we performed an MTT colorimetric assay (Figure 3b). C2C12 murine myoblasts were seeded on standard and LS plates, and their growth was followed for 72 h (Figure 3c). We observed that the MTT values obtained on the LS plates (blue line) were lower than those of the standard ones (red line), a difference which can be attributed to the reduction in the total area available for cell adhesion due to the presence of the hydrophobic laser‐induced channels. To verify this hypothesis, a theoretical curve was calculated by normalizing the values obtained with the standard plates according to the percentage of area available on the LS surfaces. The resulting values (dashed blue line) confirmed the biocompatibility of the laser‐structured surfaces, as they closely match the experimentally obtained results (blue line, Figure 3b).

#### 3.2.2 Cell Guidance and Organization

Once the safety of LS surfaces was verified, we investigated their ability to drive cell organization via contact guidance. C2C12 cells were cultured on standard and LS plates (Figure 4a–c), testing the four channel spacing values (75 µm, 100 µm, 125 µm, and 150 µm center‐to‐center) already used for contact angle analysis. The resulting myotubes were analyzed after 7 days of differentiation.

**FIGURE 4:**
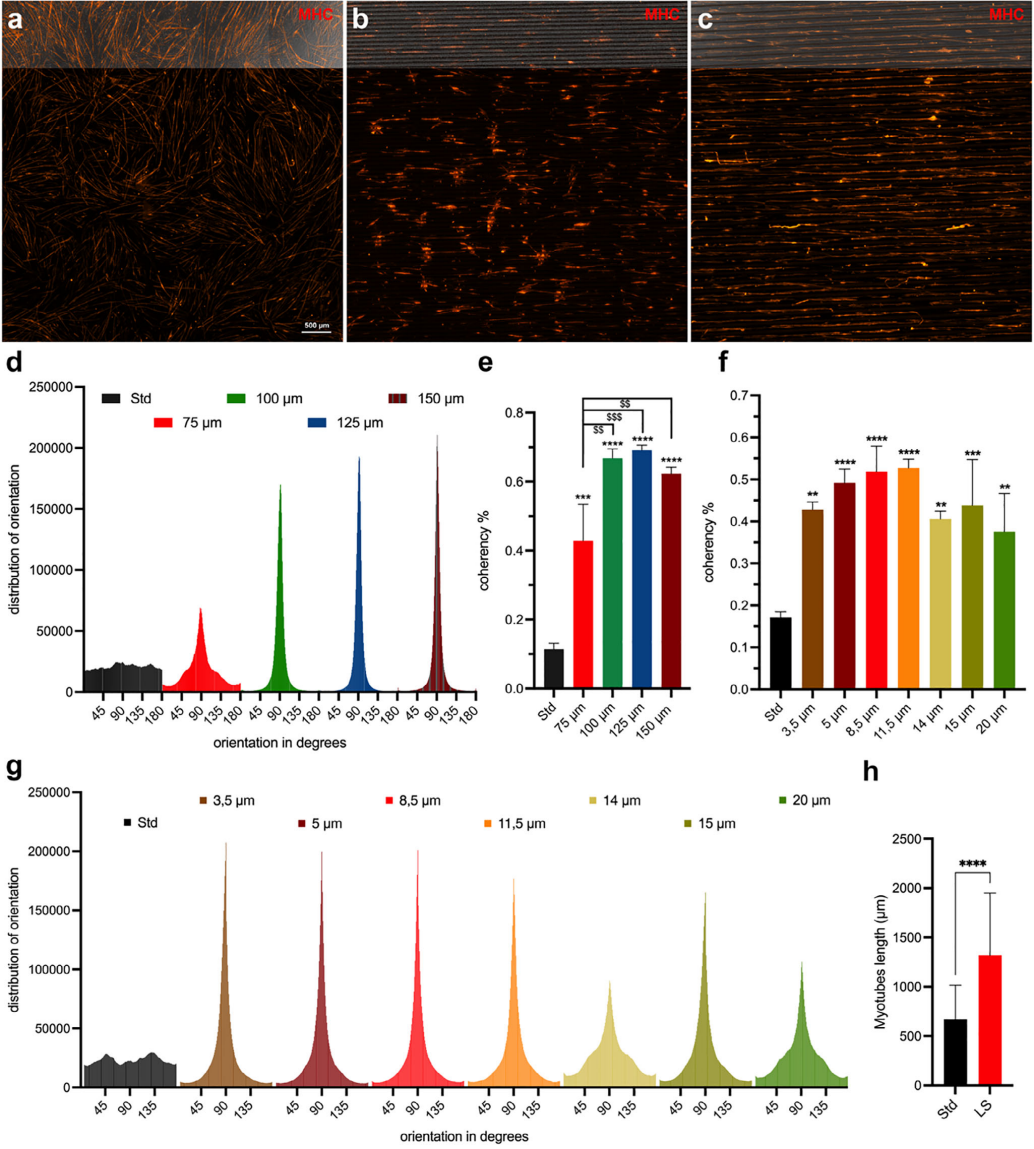
Image analyses conducted on C2C12 after 7 days of differentiation. Pictures a, b, and c show representative immunofluorescence pictures superimposed on the top with their relative brightfield pictures. The myotubes positive for MHC (Red), are randomly oriented in the standard plates (a) while properly aligned on the LS surfaces (channel spacing of 75 µm (b) and 150 µm (c)). Orientation of muscle fibers. The distribution of orientation is plotted for unstructured standard plates (Std) and for all spacing (d, 75, 100, 125, and 150 µm) and depth (g, 3.5 – 5 – 8;5 – 11.5 – 14 – 15 ‐20 µm) values tested, revealing the presence of a prevalent peak for all conditions compared to their respective control (black plot); the quantification of the orientation through the coherency value (e, f) shows a significative increase for all the LS surfaces compared to the standard (^*^). Moreover, regarding the spacing analysis, the three higher values are also significative compared to 75 µm (e,^$^). Myotube lengths. The myotubes differentiated on LS surfaces with a spacing of 125 µm and a depth of 11.5 µm (h, red bar) are significantly longer than those grown on the standard plates (h, black bar). *Sample size n* = 3. *p value* list: spacing, ^****^ *p* < 0.0001, ^***^ *p* = 0.0001, ^$$$^ *p* = 0.0006, ^$$^ *p* = 0.0013 (75 µm vs 100 µm), ^$$^ *p* = 0.0059 (75 µm vs 150 µm); depth, ^****^ *p* < 0.0001, ^***^ *p* = 0.0007, ^**^ *p* = 0.001 (3.5 µm), ^**^ *p* = 0.0025 (14 µm), ^**^ *p* = 0.0085 (20 µm); length, ^****^ *p* < 0.0001.

On the LS surfaces, cells tended to elongate along the interspace between adjacent channels rather than within the channel itself. Considering the channel width, the 75 µm spacing produced a densely packed hydrophobic pattern that strongly constrained cell development. As a result, cells formed aggregates with shorter and less organized myotubes that often crossed the channels (Figure 4b). Conversely, increasing the spacing between channels improved the control over myotube organization, providing sufficient space for them to expand in both width and length while guiding their growth along the channel edges (150 µm, Figure 4c).

To support these observations, myotube organization was further studied using orientation analysis (Figure 4d). Although all four spacing conditions showed significantly higher alignment than the standard, as indicated by the coherency measurements (Figure 4e), the smaller spacing (75 µm) resulted in a markedly lower coherency compared to the others. The highest coherency was achieved with 125 µm spacing, which was therefore selected for subsequent experiments. Secondly, we investigated the influence of channel depth on myotube organization. Seven depths (3,5 – 5 – 8,5 – 11,5 – 14 – 15 µm and 20 µm) were tested for their ability to promote myotube alignment. Interestingly, we found a significant increase in orientation for all seven values compared to the standard (Figure 4g), as confirmed by the coherency analysis (Figure 4f). Although not statistically significant, the highest coherency value was observed with channels of 11.5 µm depth, which was therefore selected for subsequent experiments. Finally, we observed that C2C12 cells differentiated on LS plates with a spacing of 125 µm and a depth of 11.5 µm formed significantly longer myotubes, more than twice as long, than those on standard plates (Figure 4h).

In addition, to assess the broad applicability of these findings, we conducted a systematic analysis of myotube orientations on LS plates from six different commercial manufacturers (Nunc, Falcon, CLEARline, SPL, Costar, SARSTEDT) encompassing formats from 6‐ to 96‐well plates as well as chamber slides. The outcomes were compared with those obtained using standard 12‐well plates (Nunc). We found that myotubes displayed improved organization compared with the standard across all tested supports (Figure S7). Furthermore, orientation analyses (Figure 5a) clearly demonstrated that the cell‐guidance effect of the LS surfaces is independent of both their brand and size (Figure 5b). All LS supports exhibited a predominant orientation peak compared to the standard plates (Figure 5a). A significant increase in coherency values was also observed for all the LS surfaces compared with the standard (Figure 5c), with no statistically significant differences among the LS surfaces themselves, thereby supporting the efficiency and universality of the structuring process.

**FIGURE 5:**
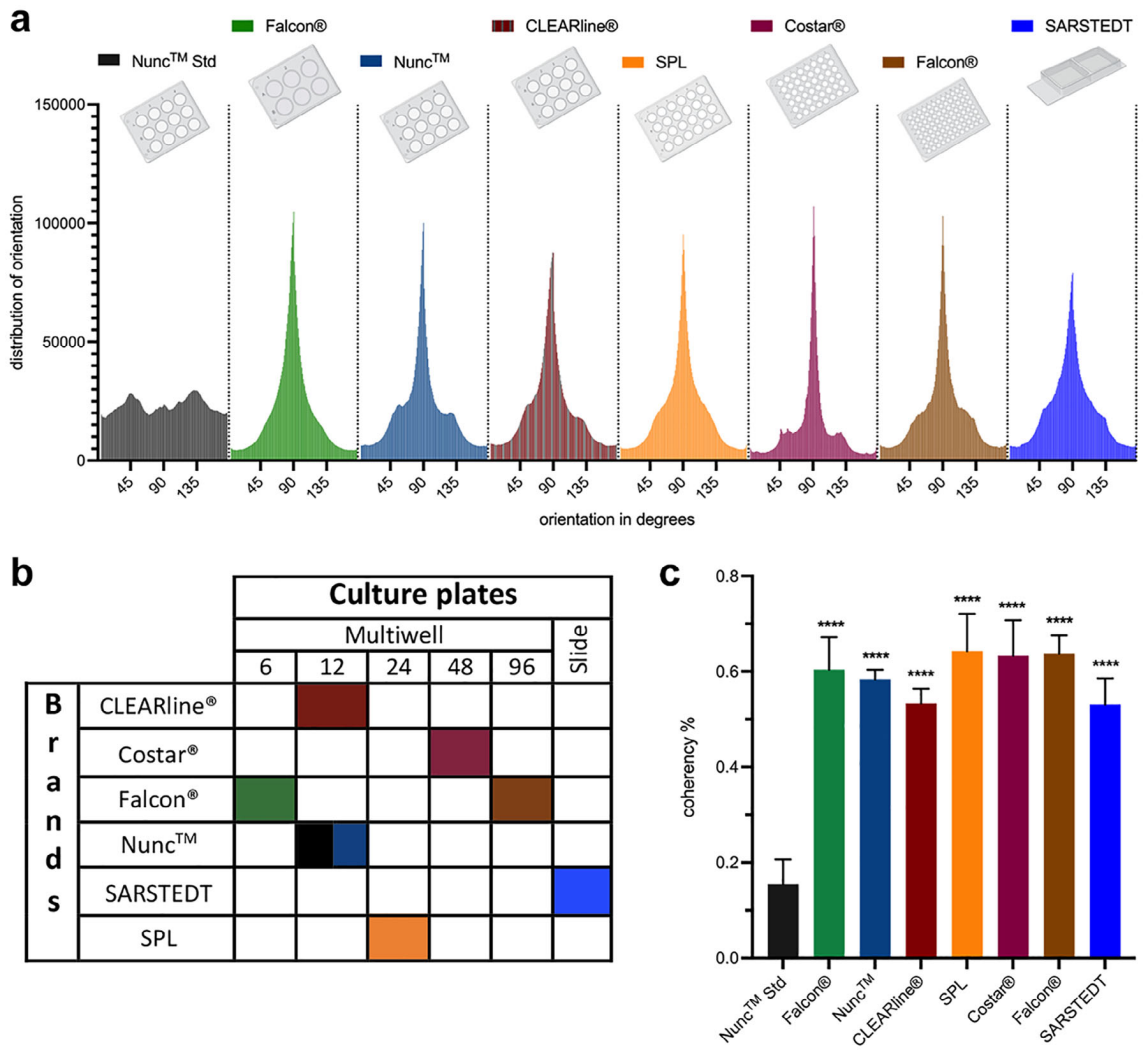
Systematic study of the effect of LS plates of different brands and sizes on myotube orientation. Distribution of orientation for all the analyzed standard (Nunc Std) and LS culture plates (a); table of the different brands and sizes of culture supports analyzed (b); Coherency values for all the analyzed samples (c) with respective significances (^*^ compared to the standard, p values: ^****^ *p* < 0.0001, *Sample size n* = 3).

It is also worth noting that the structuring process has been tested on tissue culture polymeric coverslips such as polyethylene terephthalate glycol (PET‐G, Sarstedt). These materials, being similar to polystyrene in terms of chemical composition, obtained analogous results regarding channel formation and cell alignment (Figure S8).

Finally, we tested different laser‐induced topographies, such as concentric circles and squares, and zig‐zag lines with different angles, observing a clear tendency for cells to organize themselves according to the designed patterns (Figure S9).

#### 3.2.3 Gene Expression Profiling by RT‐qPCR

The influence of LS surfaces on myoblast gene expression was further evaluated by RT‐qPCR analysis (Figure 6). The expression of genes associated with myoblast differentiation and maturation was analyzed in C2C12 cells cultured on standard and LS plates. The results showed that LS surfaces did not significantly affect gene expression during the early and intermediate stages of differentiation, including days 0 (Figure 6a), 4 (Figure 6b), and 7 (Figure 6c). In contrast, at the late stage of myotube maturation (day 11; Figure 6d), cells cultured on LS plates exhibited a significant upregulation of key myogenic markers. Increased MyoD expression suggests enhanced myogenic differentiation, while elevated Desmin and MHC levels indicate improved structural organization and muscle maturation. Furthermore, the upregulation of Myomaker reflects enhanced myoblast fusion capacity, also confirmed by increased C‐met expression, a gene with a dual role in skeletal muscle involved first in myoblast motility and later in myocyte fusion [41].

**FIGURE 6:**
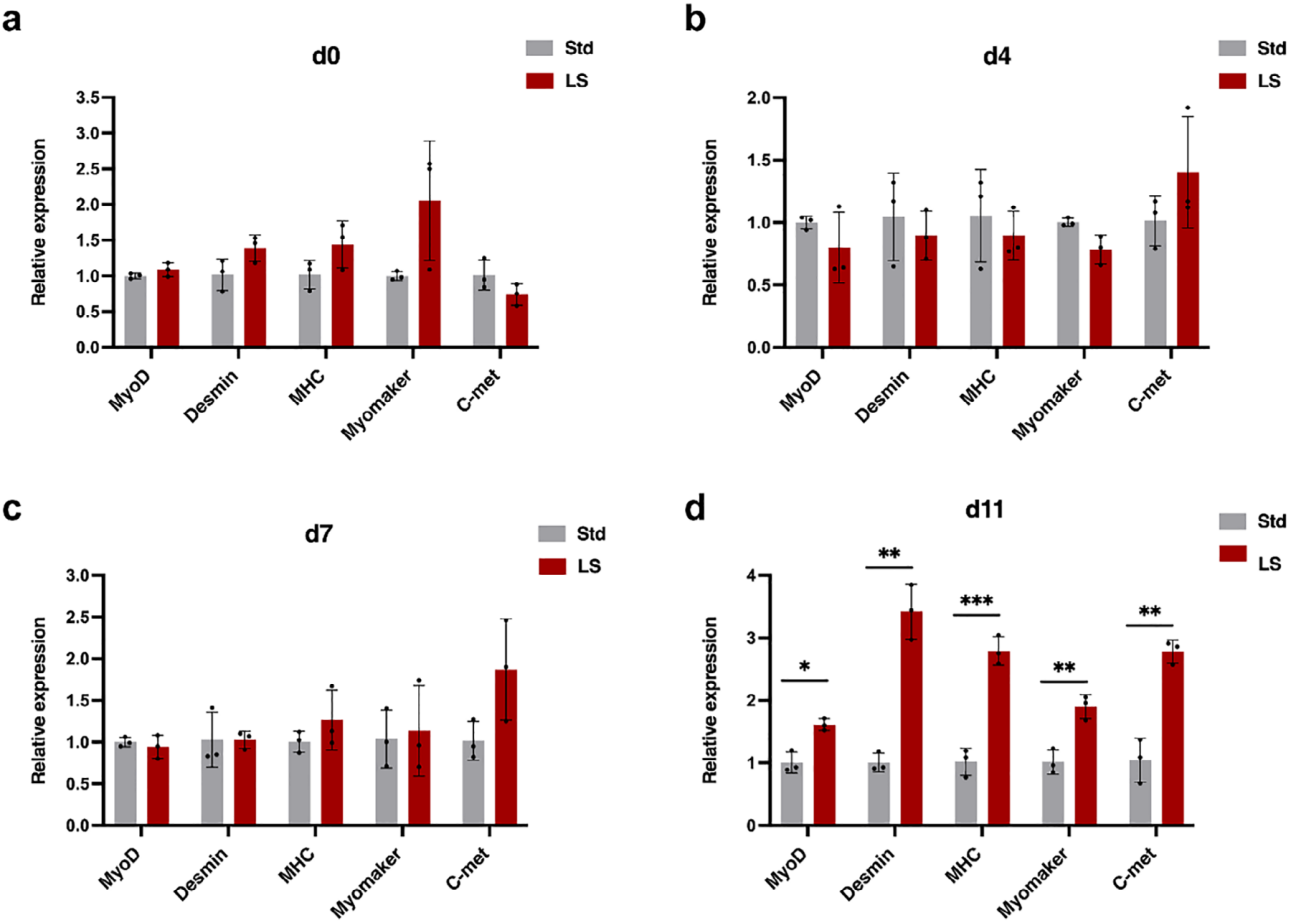
Relative gene expression of C2C12 myoblasts cultured on the LS surface compared with the standard surface. Data are presented as mean ± SD with individual data points (*n* = 3). Gene expression levels were normalized to the housekeeping gene and expressed as fold change relative to the standard group (Std). Panels correspond to: (a) d0, (b) d4, (c) d7, and (d) d11. Statistical significance was determined using Student's *t*‐test: ^*^ *p* < 0.05; ^**^ *p* < 0.01; ^***^ *p* < 0.001 vs. Standard.

#### 3.2.4 Protein Expression: Western Blotting

To complement the gene expression analysis, MHC and Desmin protein levels were evaluated (Figure 7). MHC and Desmin were selected as key markers of muscle maturation. MHC reflects the development and differentiation of the contractile apparatus, whereas Desmin is involved in maintaining the structural organization and mechanical stability of muscle cells.

**FIGURE 7:**
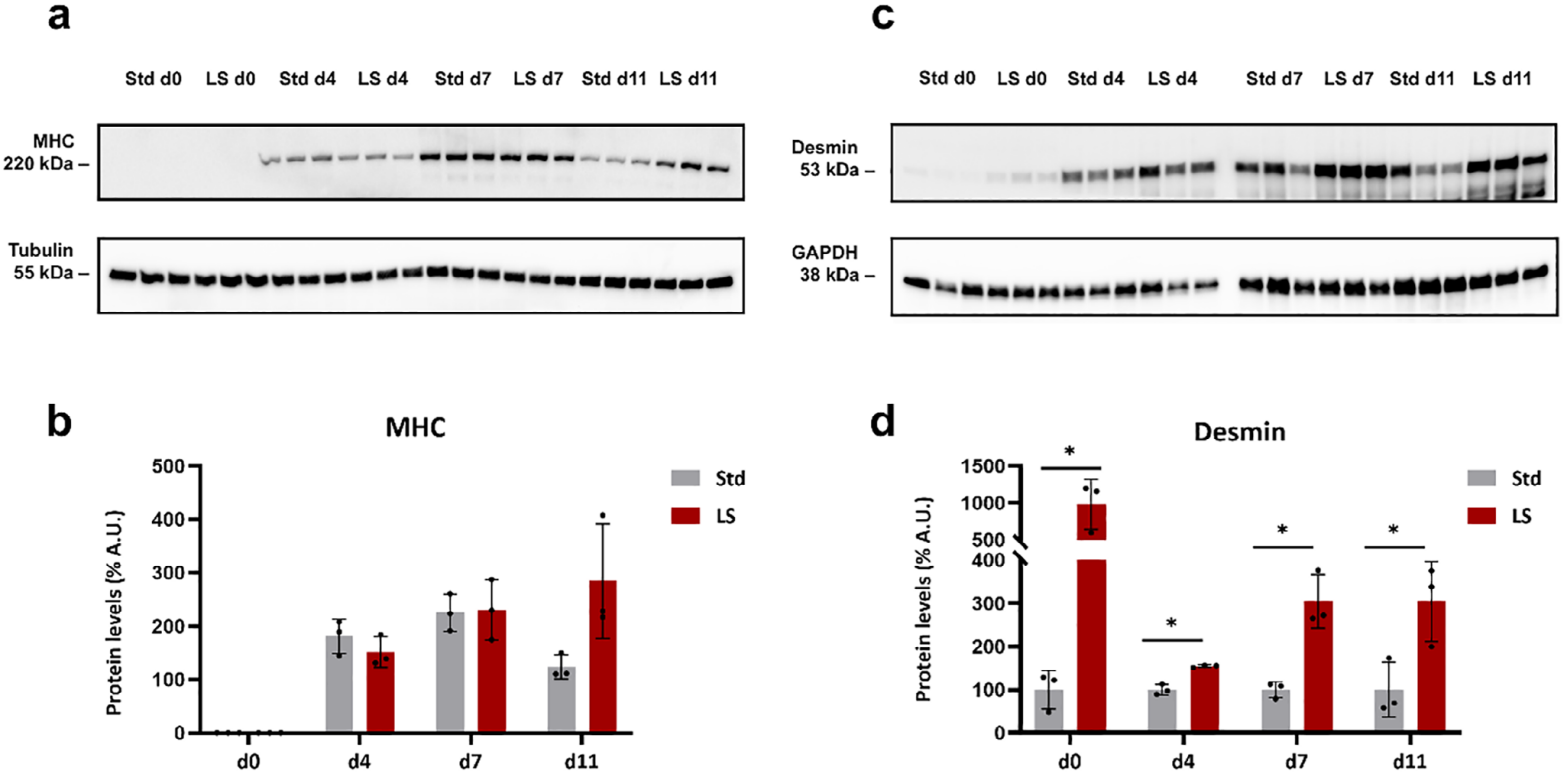
Analysis of MHC and Desmin protein levels during C2C12 myoblast differentiation. Western blot analysis of C2C12 cells cultured on standard (Std) and LS surfaces at different stages of differentiation. Representative immunoblot for (a) MHC and Tubulin, and (c) Desmin and GAPDH. Densitometric analysis of (b) MHC and (d) Desmin protein levels. Data are presented as mean ± SD with individual data points (*n* = 3). Statistical significance was assessed using Student's *t*‐test (^*^ *p* < 0.05; ^**^ *p* < 0.01; ^***^ *p* < 0.001 vs. Standard at each time point).

Although no statistically significant differences were observed, MHC levels of cells cultured on LS plates showed a tendency to increase at late stages of myotube differentiation (Figure 7a,b). In contrast, Desmin levels increased across all time points on LS plates (Figure 7c,d), suggesting enhancement of myotube organization throughout the differentiation process. Overall, these results are consistent with gene expression data, indicating that LS surfaces mainly promote late‐stage myotube maturation rather than early myogenic commitment. This supports the role of LS surfaces in enhancing structural stabilization and maturation of skeletal muscle cells, highlighting their relevance for skeletal muscle tissue engineering applications.

### 3.3 Morphological Characterization of Human Muscle Cell Cultures Under Pathological Conditions

Once we had demonstrated the ability of LS surfaces to guide muscle cell cultures, improving not only their organization but also their subsequent differentiation, we decided to apply this methodology to investigate morphological differences in myofibers associated with neuromuscular diseases. Immortalized myoblasts from healthy donors and DMD and FSHD patients were cultured on LS supports and analyzed after 7 days of differentiation.

Immunofluorescence analysis on healthy samples revealed well‐differentiated myotubes positive for MHC and Titin, which appeared highly elongated and well aligned (Figure 8a). Volumetric image reconstructions highlighted the sarcomeric organization and spatial arrangement of the myotubes relative to the laser‐induced channels (Figure 8b,c), with evenly distributed nuclei.

**FIGURE 8:**
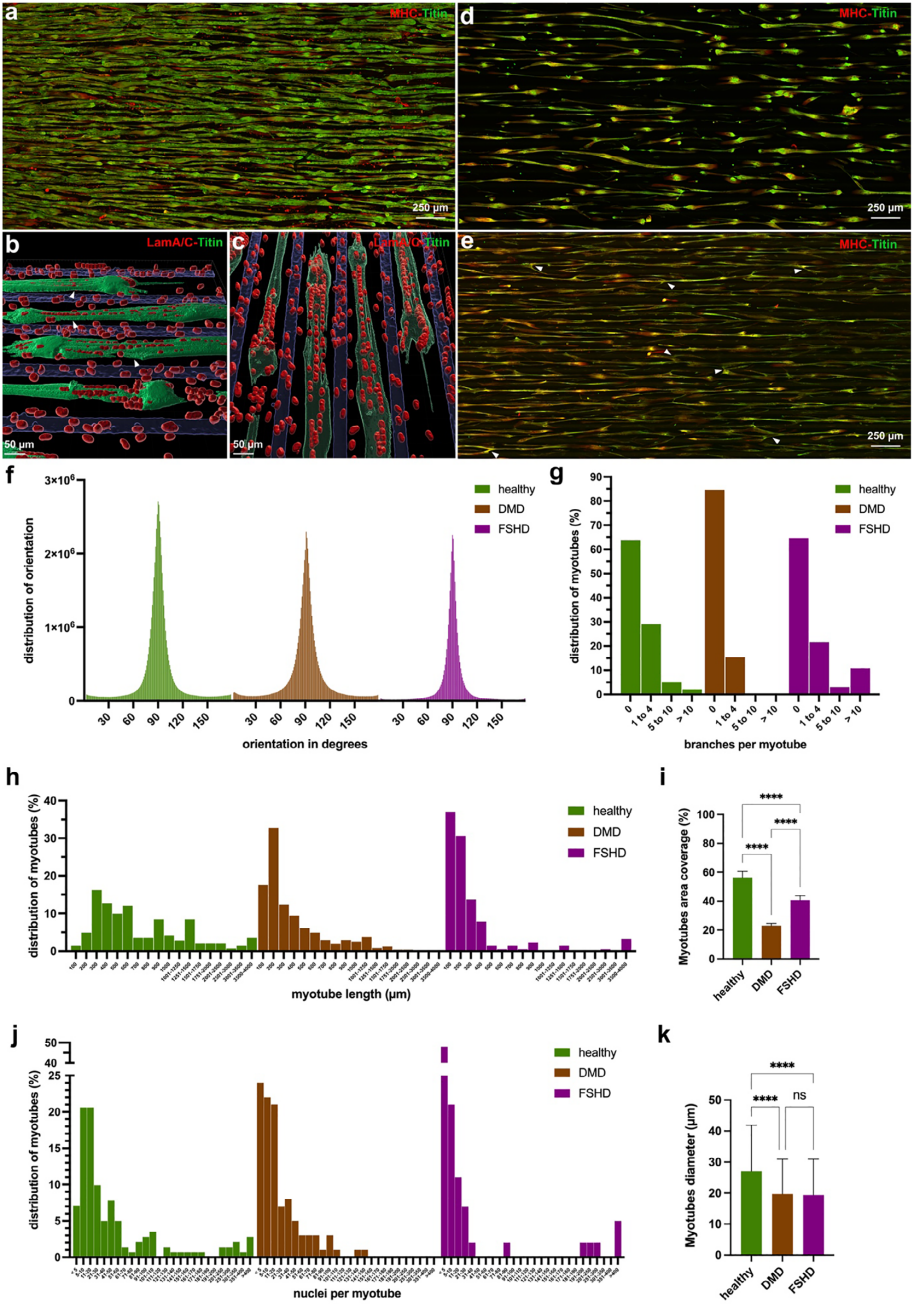
(a) Immunofluorescence analysis of healthy human myotubes stained for MHC (red) and Titin (green). Volumetric reconstruction highlighting sarcomeric structures (Titin in green, white arrowheads) – shown in solid (b) and transparent (c) shading, and nuclear organization (Lamin A/C in red). Laser‐induced channels are marked in blue, emphasizing the preferential alignment of myotubes between them. DMD (d) and FSHD (e) human myotubes stained for MHC (red) and Titin (green). Examples of branches are highlighted by white arrowheads (e). (f‐k) Healthy (green), DMD (brown), and FSHD (purple) myotube analyses: (f) myotube orientation, (g) distribution of branches per myotube, (h) myotube length distribution, (i) myotube area coverage, (j) distribution of nuclei per myotube, (k) myotube width. (*Sample size n* = 3, *p* values: ^****^ *p* < 0.0001).

The same immunostaining was performed on samples derived from DMD (Figure 8d) and FSHD (Figure 8e) myoblasts, two distinct types of muscular dystrophy. These samples also exhibited elongated, well‐organized myotubes with an orientation comparable to that observed in the healthy ones (Figure 8f). However, the three conditions exhibited distinct phenotypes and were therefore further characterized through image analysis (Figure 8g–k). Particularly noteworthy is the single‐fiber–like arrangement of the cultured myotubes, which allowed the contours of individual myofibers to be clearly distinguished and enabled a comprehensive set of morphological analyses to be performed. The area coverage, defined as the portion of the image occupied by myotubes, was significantly higher in the healthy samples compared to FSHD and DMD ones (Figure 8i). By testing the myotube length distribution, describing the range, frequency, and variability of myotube sizes observed in experimental conditions, we observed that healthy myotubes are widely distributed, with lengths reaching up to 4 mm and a majority of myofibers ranging from 300 µm to 600 µm. DMD myotubes were shorter, with a maximum length of 2.5 mm and a majority ranging from 200 µm to 600 µm while FSHD myotubes were mainly distributed between 200 µm and 400 µm but exhibited a distinct peak in the longer range (up to 3.4–4 mm, Figure 8h). In addition, the myotube width revealed a significant decrease in the diameter of myotubes for both affected cell lines compared to healthy ones (Figure 8k).

Finally, the distribution of nuclei per myotube, used as an indicator of myotube maturation, revealed the presence of highly multinucleated myotubes across all conditions, suggesting a generally higher level of maturation (Figure 8j). However, the healthy samples showed the lowest proportion of immature myotubes (<5 nuclei, 7% of the total myotubes), with a peak for myotubes containing between 5 and 20 nuclei (∼40%), a gradual decline in the number of myotubes containing up to 180 nuclei/myotube, and a slight increase in the number of myotubes containing 201 to >400 nuclei. In contrast, DMD samples showed a high number of immature myotubes (∼24%), with around 40% of myotubes containing approximately 20 nuclei, while the number of myotubes containing more nuclei (up to 150 nuclei/myotube) progressively declined. In FSHD, we observed a higher proportion of immature myotubes (∼45%), with around 20% of myotubes between 5 and 10 nuclei and a sharp decline up to 40 nuclei/myotubes. We also observed a small proportion of myotubes containing between 81–90 and 191–300 nuclei per nuclei, with a peak at >400 nuclei/myotubes (5%).

We next quantified the distribution of branches per myotube, as an indicator of branching that occurs predominantly between parallel myotubes (Figure 8g). We observed distinct patterns among samples. FSHD cultures exhibited a dense network of interconnections formed by thin branches extending between adjacent myotubes (Figure 8e). Quantitative analysis showed that although the overall distribution of branches per myotube was as broad as in healthy samples, FSHD cultures contained a higher proportion of myotubes with more than 10 branches. In contrast, DMD myotubes were largely unbranched (85%), with the remaining fraction displaying low levels of branching (1–4 branches).

Interestingly, the FSHD samples displayed the highest proportions of nuclei and branches per myotube in the upper ranges of the measured distributions (Figure 8j,g), two findings that are mutually reinforcing. Notably, the extensively branched myotubes formed complex superstructures that integrated a remarkably large number of nuclei.

## 4 Discussion

Several solutions are commercially available to obtain organized and aligned 2D cultures, mostly based on complex manufacturing methods and requiring additional treatments or coatings to enable cell adhesion. Although effective for obtaining organized cell cultures, these methods force researchers to use a limited range of cell culture supports, making their use less flexible and accessible to the scientific community.

Here, we thoroughly investigated the capacity of a laser‐based structuring process to create micrometric topographic cues on the surface of standard, commercially available cell culture supports. We then evaluated and analyzed their associated ability to drive muscle cell orientation and differentiation via contact guidance. The use of a high‐speed scanning system allowed us to precisely control the patterning design in a fast and efficient manner. At the same time, the use of an ultra‐fast laser minimized the energy required to modify PS surfaces, consequently reducing induced thermal effects and subsequent generation of possible toxic compounds (Figure 3a). We further showed that the fabrication process proposed here provides effective, highly reproducible, and cell‐safe supports for highly organized cell culture systems, with a focus on anisotropic 2D cell patterns applied to skeletal muscle modeling. However, this innovative cell culture system remains a 2D platform, as it does not recapitulate the 3D organization and biochemical composition of the native extracellular matrix of skeletal muscle.

Given the results presented in the preceding section, several key aspects of the proposed work merit further in‐depth discussion.

### 4.1 Physical Characterization of Polystyrene LS Surfaces

As it is the case for many other polymers, solid polystyrene exhibits extensive absorption up to 350 nm due to the formation of charge‐transfer contact complexes with oxygen [42, 43]. Naturally, numerous studies have focused on the UV laser irradiation of polymers for patterning approaches, micro/nano structuration, and tunable wettability modification. Depending on laser parameters, the ablation of PS can be done through several mechanisms based on photochemical and photothermal phenomena [21, 29]. The use of an IR femtosecond laser limits the ablation of the polymeric surface to photothermal mechanisms with limited photochemical effects and photodegradation [44], as Wang and colleagues demonstrated [45] by simulating the interaction of a 795 nm 130 fs laser with a PS surface. They showed a large percentage of laser energy converted into heat, which dissipated in the bulk of the material, induced plasma generation, and ablation of the material itself, corroborating our observations. Topography is created here through ablation by vaporization and melting of the PS surface, leading to the ejection of the targeted material. Suriano and colleagues [21] irradiated PS samples with 800 nm femtosecond pulses up to 44.2 J/cm^2^. At such intensity, they reported a substantial darkening of the PS. These changes in appearance were explained by oxidative degradation of the polymer under strong laser irradiation. They finally confirmed this hypothesis by displaying the evolution of the IR PS absorbance during µ‐ATR‐FTIR spectrum analysis depending on the incident laser fluence, which, for high fluence, indicated the appearance of ─OH (3400–3600 cm^−1^) groups and broad bands due to carbonylic species (1700 cm^−1^). In our case, laser fluence is limited to 2.3 J/cm^2^ at 1030 nm, and, given the obtained IR absorbance spectra of the treated surfaces and further corroborated by the lack of the C═O group absorption band at 1730 cm^−1^, usually associated with photodegradation [46], we can confidently assume that limited oxidation was generated by our process. The darkening of the observed channels is here only due to the creation of a highly porous surface. In this regard, the increased surface area associated with the porosity created by laser irradiation amplifies the number of exposed non‐polar functional groups and, thus, the hydrophobicity of the polystyrene [47]. This effect is further amplified by the removal of the culture plate surface treatment and, as demonstrated by Wang and colleagues, by the potential chemical bond modifications within the polymer at low fluences [48].

### 4.2 Topographical Cues for Contact Guidance

We demonstrated that introducing a partial topographical modification on a standard PS plate surface (parallel lines spaced from 75 µm to 150 µm) generated hydrophobic areas that act as barriers, as evidenced by the differences in contact angles between standard and LS plates. In addition, the deep channels and protrusions formed through PS melting and redeposition during the laser‐structuring process further enhanced these physical cues, creating a topographical environment highly conducive to contact guidance. Experiments with murine myoblasts confirmed the effectiveness of the LS plates in driving cell adhesion and orientation, hence promoting the alignment of the resulting myotubes (Figure 4). The different channel spacing tested highlighted the underlying mechanisms influencing cell organization. Closely spaced hydrophobic channels do not provide enough suitable areas for proper cell adhesion and growth, resulting in a loss of alignment and the formation of cell clusters. In contrast, wider channel spacing allowed sufficient space for cell development, promoting better alignment and organization. The improved, large‐scale organization also led to a significant increase in the average length of myotubes when compared to those grown on standard, unstructured support. These results showed that the anisotropic environment promoted more efficient surface utilization, with myotubes that, when guided to elongate in a preferential direction, reached at least twice the length of those in conventional culture conditions.

Gene and protein expression data (Figures 6 and 7) indicate that LS surfaces promote late‐stage myotube maturation. The overexpression of MyoD mRNA at day 11 may reflect the persistence of a subpopulation of cells that remains in an active differentiation state compared with the control condition. At the same time, the upregulation of the other myogenic genes combined with the increased levels of Desmin and MHC proteins suggests that myotubes differentiated on LS surfaces reach a more advanced stage of maturation. Interestingly, the geometrical and physical constraints induced by LS surfaces promote cell alignment from the proliferation stage onward (Figure 3c), and this effect is associated with an early increase in Desmin protein levels, likely as an adaptive response to topography‐induced mechanical stress [49]. As differentiation progresses, the statistically significant increase in Desmin levels observed at each time point suggests the development of a more organized and mature sarcomeric architecture.

We also ensured that the observed results were not related to a specific brand or type of support (Figure 5; Figure S7). To this end, the structuring process and subsequent cell analyses were repeated on commonly used culture plates, ranging from 96‐ to 6‐well formats, as well as on chamber slides and treated polymeric coverslips. We obtained significantly higher alignment values for each tested sample compared to the standard unstructured plates. On the other hand, we observed no significant differences between the brands and sizes tested. Altogether, these results demonstrate the validity and versatility of the laser structuring technique to functionalize polymeric cell culture substrates. The proposed laser‐structuring technique is thus an effective and reliable solution for the large‐scale production of micro‐structured plates for standardized and highly organized cell cultures.

### 4.3 Characterization of Autofluorescence and Solutions

An inherent side effect of the laser‐structuring process was the appearance of autofluorescence. As it is well documented in the literature, mechanical and thermal modifications of polymers can lead to its increase, and this phenomenon could therefore limit the range of applications of LS surfaces, especially for immunofluorescence analysis [37]. This aspect was thoroughly investigated to define a strategy to reduce, and ideally eliminate, its impact on the use of LS PS surfaces.

Polystyrene is a synthetic aromatic polymer with monomer units linked by covalent bonds to form macromolecular chains [50]. Its chemical composition and, notably, the presence of aromatic benzene rings lead to some degree of intrinsic fluorescence [51]. This fluorescence can emanate from monomer emission with a fluorescence peak at 280–285 nm or excimer emission at 335 nm [52]. The localized reconfiguration of the material and the increased surface area resulting from the formation of highly porous structures eliminate the need for any surface treatment and manufacturing processes specifically aimed at reducing the fluorescence abilities of the benzene ring. The increase in fluorescence can also be explained by the reorganization of polymer chains and the formation of excimers following direct laser writing [37]. Given the proximity of the PS autofluorescence peak to the absorption and emission spectra of blue fluorophores (e.g., DAPI), this severely limits the use of conventional nuclear counterstaining. As we observed (Figure S5) and as confirmed by Young and colleagues [37], the intensity of the autofluorescence decreases at longer wavelengths. In this context, one possible solution is to use fluorophores with spectra distant from the affected channel. However, this inevitably limits the number of markers that can be visualized simultaneously within the same sample. Alternatively, confocal microscopy proved to be a straightforward and widely accessible method for overcoming the autofluorescence associated with LS plates. The autofluorescent signal originates primarily from the laser‐structured channels and exhibits minimal spread along the z‐axis. When combined with the high depth‐resolution capabilities of confocal imaging, this substantially reduces autofluorescence interference and enables reliable fluorescence acquisition for all fluorophores (Figure 8). Both approaches are effective but do not address the underlying issue. To eliminate, or at least reduce, the autofluorescence of LS surfaces, we therefore explored the use of photobleaching. Photobleaching is a form of photodegradation in which fluorescence gradually diminishes upon prolonged exposure to light, particularly at high intensities. This process occurs because the energy absorbed by the fluorescent molecules within the polymer (the benzene ring for PS) can cause chemical reactions, such as bond breakage or the formation of new, non‐fluorescent chemical species. These changes degrade the molecular structure that originally emitted fluorescence, leading to a decrease in the overall fluorescence intensity over time. In the case of PS, exposure to light, especially at UV wavelengths, leads to the creation of radicals whose nature depends on the spectral composition of the light, and to overall photooxidation [53]. Hawkins and colleagues [54] reported a decrease in PS autofluorescence under continuous illumination at a wavelength between 500 nm and 645 nm. The observed nonlinear decrease joins the observations of Piruska and colleagues [38]. Sullivan and colleagues [55] investigated the undesired loss of fluorescence in microplastics by illuminating various polymers with 405 nm light produced by a solid‐state laser. They noticed a similar nonlinear curve with a drastic reduction of fluorescence (minus 70%) plateauing after 30 min exposure. Here, we leveraged this phenomenon to our advantage and achieved a comparable reduction in autofluorescence (minus 90%) by exposing the LS surfaces to 365 nm UV LEDs for 10 min. Importantly, this treatment did not adversely affect cell behavior, as evidenced by unchanged differentiation and alignment capabilities (Figure S5k,l). The oxidation of the PS surface and associated rearrangement of the benzene ring due to photooxidation is a direct consequence of the UV exposure, as demonstrated by Pfleging and colleagues [56]. The use of LEDs allows for an accessible and affordable solution, and their broad illumination guarantees the quick processing of structured commercial culture plates. Therefore, the combination of efficient photobleaching and confocal microscopy markedly reduces the unwanted autofluorescence induced by the laser‐structuring process.

### 4.4 Morphological Characterization of Human Myofibers

Nowadays, there is increasing pressure to reduce and even replace animal testing, motivated by its inadequacy in replicating the pathophysiological aspects of human diseases. The issue is underlined by the failure of over 90% of new drugs tested on animals during clinical trials [57], making the need to develop reliable human disease models even more evident. Therefore, we explored the potential of LS surfaces as an advanced 2D culture system for the morphological characterization of myotubes under both physiological and pathological conditions. Our aim was to set up a human‐based platform suitable for precise image analyses, enabling the discrimination of disease‐induced phenotypical differences. We focused on two of the most frequent neuromuscular diseases, Duchenne muscular dystrophy (DMD) and Facioscapulohumeral dystrophy (FSHD), and obtained organized skeletal muscle cell cultures (Figure 8) able to replicate morphological features characteristic of the diseases studied. Overall, analysis of the myotube area coverage (Figure 8i) revealed a reduced myogenic capacity in pathological samples compared with healthy controls, attributable to factors such as fewer myotubes, smaller myotube size (length and/or width), or both. This parameter provides a general snapshot of their pathological state. More in‐depth analyses showed, for example, that FSHD samples exhibited thin, elongated, and highly branched myotubes (Figure 8e,k,h,g), a phenotype expected for this disease [58, 59]. Similarly, DMD myotubes appeared smaller and more immature than healthy ones (Figure 8d,k,h,j), consistent with the typical DMD phenotype [60]. Importantly, due to the contact guidance effect of the LS surfaces, the muscle models exhibited a high degree of organization, with highly elongated myotubes perfectly aligned along the anisotropic topography (Figure 8b,c,f). This alignment enabled accurate analyses of myotube morphology in a single‐fiber‐like context, in which individual myotubes could be clearly distinguished and isolated from the surrounding culture. This represents a major advantage of the proposed system and a substantial improvement over conventional 2D culture approaches.

Altogether, (i) the ability to apply the laser‐structuring process to virtually any cell culture substrate without disrupting standard workflows, (ii) the high degree of organization and enhanced differentiation achieved in the resulting muscle cultures, and (iii) the quantity and quality of image analyses they enable, collectively open the way to a broad range of applications, from high‐resolution microscopy of individual myotubes to high‐throughput drug screening.

## 5 Conclusions

Here, we report the development, characterization, and validation of a micrometric laser‐structuring process designed to functionalize standard culture plates, specifically tailored for skeletal muscle modeling. The creation of topographical cues through laser ablation constitutes a rapid, reliable, and reproducible digital process applicable to a wide range of cell culture supports, with virtually no limitations on the size or complexity of the desired LS pattern. The versatility of this methodology allows seamless integration into diverse experimental designs and easy adaptation to researcher‐specific requirements. Importantly, the direct modification of standard plates eliminates the need for time‐consuming and costly mold fabrication, as required in techniques such as soft lithography or melt‐casting. This innovative approach could readily be adapted to reproduce the characteristic topographies of other tissues and organs, extending the potential applications of this 2D culture system beyond skeletal muscle modeling.

## Author Contributions

**Lucas Duvert**: conceptualization, methodology, writing – original draft, writing – review and editing, investigation, formal analysis, data curation. **Lorena Garcia‐castillo**: writing – review and editing, formal analysis, methodology, investigation. **Clarissa Murru**: methodology, formal analysis, investigation. **Adrien Casanova**: funding acquisition. **Daniel Ferry**: methodology. **Pierre Perrin**: methodology. **Solene Coste**: methodology. **Anne Bigot**: resources. **Anne‐Patricia Alloncle**: funding acquisition, conceptualization, writing – original draft, writing – review and editing. **Frederique Magdinier**: writing – review and editing, writing – original draft, funding acquisition. **Stefano Testa**: conceptualization, methodology, investigation, data curation, supervision, writing – review and editing, writing – original draft, validation.

## Conflicts of Interest

The authors declare no conflict of interest.

## Supporting information




**Supporting File**: advs78129‐sup‐0001‐SuppMat.docx.

## Data Availability

The data that support the findings of this study are available from the corresponding author upon reasonable request.
