# Supplementary material for: Ultrafast Micrometric Laser Structuring of Polymer Surfaces for Highly Organized Skeletal Muscle Tissue Modeling
Source: Adv Sci (Weinh). 2026 Oct 4:e78129. Online ahead of print. doi: 10.1002/advs.78129 (PMC13634577; doi:10.1002/advs.78129)
Supplement: Supplementary file 1 [file ADVS-9999-e78129-s001.docx]

**Supplementary figures**

**Supplementary Figure 1** Experimental set-up for laser structuring.

**Supplementary Figure 2** (**a**) Profile lines for depth and width measurement of the LS channels on polystyrene (PS) standard surfaces. (**b**) Topographical image of the channel. Clear protrusions can be seen on the side of the channel with an indent of the PS surface in the center. (**c**) Detailed measurement of the channel depth, width and height of the protrusions.

**Supplementary Figure 3:** Influence of porosity on autofluorescence. Channels of similar dimension (18µm depth) were created on standard plates using laser pulses of 213 femtosecond (**a, top**) and 10 picoseconds (**a, bottom**). Rugosity was visualized by confocal microscopy and two key parameters (Rq mean roughness and RPc peak count number) were reported. (**b**) porosity increases with the use of femtosecond pulses. The associated autofluorescence of the channels are displayed in (**c**). Measured mean grey value (MGV) and grey value (GV) are reported in the table (**d**). We can notice a reduction in MGV with lower porosity channels.

**Supplementary Figure 4:** Photobleaching effect on standard plates after 10 minutes of continuous microscope exposure at the DAPI wavelength. For each fluorescence channel (DAPI–**a**, GFP–**d**, CY3–**g**, and CY5–**j**), a 3D visualization of fluorescence intensity is shown (**b**, **e**, **h**, **k**). The corresponding gray value intensity profiles across the samples are also reported (**c**, **f**, **i**, **l**). Each peak represents a fluorescence signal from a specific channel, with intensity decreasing at the center, corresponding to the illuminated spot (**a**, dashed line).

**Supplementary Figure 5:** Photobleaching device to reduce PS autofluorescence after laser-structuring based on UV LEDs (**a,b**). Associated light spectra of UV-LEDs (**c**) are displayed in comparison with the absorption spectra of PS (**d**). Autofluorescence signal for Dapi (**e**), GFP (**f**), CY3 (**g**), and CY5 (**h**) wavelengths just after laser structuring of standard plates. Curve of the autofluorescence values for Dapi calculated over time during the continuous exposure to UV LEDs (**i**). Autofluorescence values for Dapi, GFP, CY3 and CY5 calculated just after laser structuring (LS) and after 10 minutes of exposure to UV LED with a wavelength of 365 nm (LS-PB (UV), **J**). Fusion index (**k**) and distribution of orientation (**l**) calculated on C2C12 cells after 7 days of differentiation on standard (Std), LS and LS-PB surfaces.

(p values: **** p < 0.0001, n=3).

**Supplementary Figure 6:** Contact angle analysis. Scheme of the experimental settings (**a**); side view at 0° and 90° for the standard (Std, **b,d**) and laser-structured (LS, **c,e**) surfaces; Top view of the droplet on Std (**f**) and LS (**g**) surfaces; histogram of the contact angle values for the different conditions at both 0° and 90° (**h**) with their relative significance compared to the respective control (Std, $) and between each experimental group (*). (p values: **** p < 0.0001, ^$$$$^ p < 0.0001).

**Supplementary Figure 7:** Immunofluorescence analysis for all the analyzed brands and types of standard plates. The pictures represent the myotubes, positive for Myosin Heavy Chain (MF20 antibody, Red), with the juxtaposition of the respective bright-field pictures (light gray) to visualize LS channels. Standard unstructured 12-well plate Nunc^TM^ (**a**), LS 6-well plate Falcon® (**b**), LS 12-well plate Nunc^TM^ (**c**), LS 12-well plate CLEARline (**d**), LS 24-well plate SPL (**e**), LS 48-well plate Costar® (**f**), LS 96-well plate Falcon® (**g**), LS chamber-slide SARSTEDT (**h**). Scale bar = 150 µm.

**Supplementary Figure 8:** Characterization of the laser structuration process on PET-G (polyethylene terephthalate glycol-modified) cell-adhesion treated coverslips. SEM images of the laser-ablated channels (**a**); topographical characterization of the created channel and porosity (**b**). Dimension of the ablated channels, the different dimensions match the one observed on PS (**c**). Murine C2C12 myoblast alignment on unstructured (ctrl) and LS PET-G surface. Strong cell alignment can clearly be observed on the LS surfaces with a single peak (**d**). Natural autofluorescence and result of photobleaching after UV-LEDs exposure, once again following the same pattern as for PS (**e**).

**Supplementary Figure 9:** complex LS patterns. The myoblasts stained for phalloidin (red) follow the LS channels (violet) along different complex patterns. Scale bar = 150 µm.
